# The effect of estrogen-containing birth control pills on the constituents of bradykinin expression in plasma

**DOI:** 10.1016/j.jacig.2024.100226

**Published:** 2024-02-12

**Authors:** Janette M. Birmingham, Juan Wisnivesky, Paula J. Busse

**Affiliations:** a Division of Allergy and Clinical Immunology, Icahn School of Medicine at Mount Sinai, New York, NY; b Division of Internal Medicine, Icahn School of Medicine at Mount Sinai, New York, NY

**Keywords:** Hereditary angioedema, bradykinin, estrogen, kininogen, oral contraceptives

## Abstract

**Background:**

Hereditary angioedema with C1-inhibitor deficiency (HAE-C1INH) is a rare autosomal disorder presenting with recurrent angioedema. Estrogen-containing medications trigger angioedema in some patients, and conversely, progesterone may decrease attack frequency. The mechanism by which estrogen may exacerbate angioedema in HAE-C1INH is not well characterized.

**Objective:**

Our aim was to investigate the link between estrogen and bradykinin constituents to better understand the specific underlying triggers that may exacerbate angioedema in patients with HAE-C1INH.

**Methods:**

As estrogen is contraindicated for patients with HAE-C1INH, females without a history of angioedema were recruited to evaluate whether estrogen-containing oral contraceptive pills (OCPs) alter plasma protein levels of bradykinin, cleaved high-molecular-weight kininogen (cHK), and activated factor XII (FXIIa). Blood (plasma) was collected before initiation of OCP administration and 3 months thereafter. High-molecular-weight kininogen (HK) was measured by ELISA and FXIIa and cHK were analyzed by Western blot analysis.

**Results:**

A total of 12 adult females without HAE-CINH (aged <40 years) had a median baseline plasma HK level of 33,976 ng/mL. After 3 months of OCP therapy, their median HK level increased to 38,202 ng/mL. With OCPs, there was also a significant increase in level of FXIIa protein (*P* <.01), as well as an increase in cHK protein level.

**Conclusion:**

This preliminary study, performed in females without HAE-C1INH, suggests that estrogen may exacerbate angioedema by increasing the production of cHK and FXIIa.

## Introduction

Hereditary angioedema (HAE) is a rare autosomal disorder secondary to a mutation in 1 allele encoding for C1-inhibitor (C1-INH).^1^ HAE presents clinically with acute episodes of angioedema, which typically occur in the abdomen, extremities, gastrointestinal tract, face, and larynx, the latter of which may be life-threatening without appropriate therapy. Many times, the precipitating triggers of angioedema are unknown, but in some patients they include trauma, surgical procedures, emotional stress, and infections. Administration of estrogen-containing medications (including combined oral contraceptives with progesterone) in patients with HAE is contraindicated, as it can exacerbate angioedema; case reports suggest that progesterone may attenuate symptoms.^2^, ^3^, ^4^, ^5^

The pathophysiology of HAE is due to excess bradykinin (BK) production, which after binding to the BK receptor B2 (BKRB2) results in vasodilation, vascular leak, and subsequent angioedema.^6^^,^^7^ Activation of factor XII (FXII) converts prekallikrein to kallikrein, and digests high-molecular-weight kininogen (HK) to produce BK and cleaved high-molecular-weight kininogen (cHK). Normally, excess BK production is regulated by several mechanisms, including (1) the production of functional C1-INH (abnormal in patients with HAE-C1INH), which limits the generation of BK from HK, and (2) conversion of BK to its inactive compounds by angiotensin-converting enzyme (ACE) and aminopeptidase-P (AP-P).^6^^,^^7^ Accurate measurement of plasma BK is challenging, as the estimated half-life of free BK in plasma is less than 30 seconds.^8^ Therefore, cHK and FXIIa have been reported as biomarkers of BK release.^9^

The underlying mechanism(s) of how estrogen exacerbates HAE are not well characterized. Studies in cardiovascular disease and coagulation suggest that estrogen could affect BK expression at several levels, including by modulating FXII gene transcription, regulating the expression of the B2 receptor expression, and decreasing BK degradation through attenuation of APP activity.^10^, ^11^, ^12^ Therefore, the objective of this study was to investigate whether constituents of BK expression in plasma are modulated with administration of estrogen-containing oral contraceptive pills (OCPs). The hypothesis was that estrogen-containing OCPs would increase markers of BK, in particular, HK and activated FXII (FXIIa). We report data from a study of female participants without HAE in whom the expression of these BK mediators was measured before and 3 months after initiation of administration of estrogen-containing OCPs.

The study cohort consisted of 12 premenopausal females, aged 18 to 40 years, who wished to start taking oral contraceptives. They were excluded if they had evidence of gynecologic disorders (such as polycystic ovarian syndrome, endometrial cancer, ovarian cancer, or breast cancer), use of any form of estrogen or progesterone in the 3 months preceding enrollment, use of angiotensin-converting enzyme inhibitors, a history of angioedema (including C1-INH deficiency and acquired C1-INH deficiency), pregnancy in the past 6 months, uncontrolled hypertension, renal failure, or a history of thrombosis or other contraindications for OCPs or if the patient was currently breast-feeding. Subjects were identified through advertisements placed locally at The Mount Sinai Hospital and in the surrounding neighborhood. The study consisted of 3 visits. At visit 1, after providing informed consent, patients underwent a brief physical examination and their medical history and medication use were collected. Subjects returned for visit 2 (between days 21 and 25 of their menstrual cycle) for a blood draw, after which they began taking OCPs as prescribed by their gynecologist. Subjects returned for a third visit 3 months later for the same blood collection as during visit 2, which has been determined to be time of steady-state of estrogen after the administration of oral estrogen-containing contraceptives.^13^

Two vials of blood were collected at both visit 2 and visit 3. To avoid artificial activation of the contact pathway system during the blood collection, plasma was collected by clean venipuncture and removal of the tourniquet following blood flow to decrease stasis. The first tube of blood was discarded to minimize activation caused by the initial needle puncture injury. The second tube of blood was collected in sample collection/anticoagulant tube protease inhibitor collection tubes for BK assays (SCAT-169, Haematologic Technologies, Inc, Essex Junction, Vt). Following collection, the plasma was separated and aliquots were frozen at –80°C.

Plasma HK level was measured using a commercial ELISA kit (Abcam, Waltham, Mass) and diluted to 1:100,000 as described in the protocol.

Plasma was thawed and immediately placed on ice. Samples were prepared using 4 μL of plasma, 50 μL of NuPAGE lithium dodecyl sulfate sample buffer (4×, lithium dodecyl sulfate with 10% basal medium Eagle) and 146 μL of double-distilled water. In addition, FXII and HK were prepared and used as positive controls, whereas FXII-deficient plasma was used as a negative control (Enzyme Research Lab, South Bend, Ind). Samples were boiled, loaded on 4% to 12% Tris-glycine gels, subjected to SDS-PAGE in NuPAGE MES SDS running buffer (Thermo Fisher, Waltham, Mass), and wet-transferred to an Immobilon-FL polyvinylidene difluoride membrane (Millipore Corporation). Membranes were probed with primary antibodies (Abcam) against FXIIa (68 KDa; 1:2000) and KNG (45 KDa; 1:10,000) with shaking overnight at 4°C. Transferrin (1:1000) was also run for normalization. Incubation with the primary antibody was followed by appropriate infrared-labeled second antibodies and detected using the Odyssey Infrared Imaging System (LI-COR Biosciences, Lincoln, Neb). Band densitometry was measured using ImageJ software and assigned an arbitrary value based on area.^14^

We compared the median values of HK, cHKm, and FXIIa at visits 2 and 3 by using the Wilcoxon signed rank test. Analyses were conducted using SAS statistical software (SAS Institute, Cary, NC) with a *P* value of .05 indicating significance.

## Results and discussion

A total of 27 participants with a median age of 26 years were recruited. In all, 12 subjects (median age 25 years) completed both visit 2 and visit 3. Those participants were taking OCPs with a level of estrogen greater than 25 μg.

**Fig 1:**
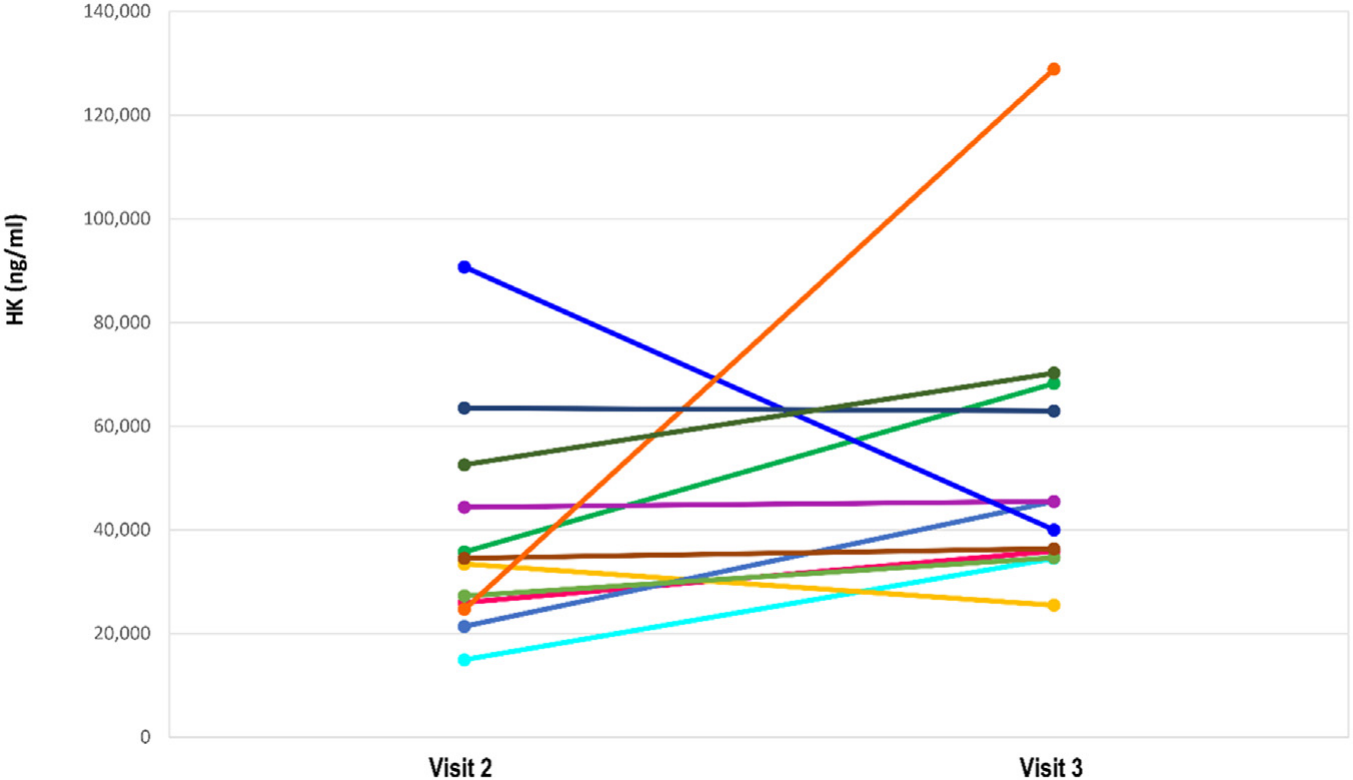
Effect of OCP use on plasma HK level at visit 2 (before OCP therapy) and visit 3 (after 3 months of OCP therapy) by ELISA. The 12 adult female participants had a median baseline plasma HK level of 33,976 ng/mL. After 3 months of OCP therapy, their median level HK increased to 38,202 ng/mL (*P* = .38). Plasma HK level was measured by using a commercial ELISA kit (Abcam, Waltham, Mass).

The 12 adult female participants had a median baseline plasma HK level of 33,976 ng/mL. After 3 months of OCP therapy, their median HK level increased to 38,202 ng/mL (*P* = .38 [Fig 1]).

**Fig 2:**
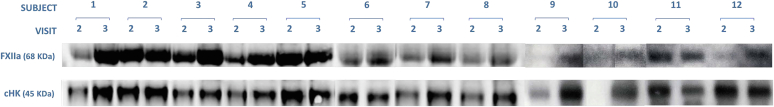
Estrogen-induced activation of the contact pathway. Western blot analysis showing increased expression of FXIIa and cHK after 3 months of administration of estrogen-containing OCPs. Plasma was collected from 12 participants at visit 2 (before OCP therapy) and visit 3 (3 months after OCP therapy). The experimental controls include HK and FXIIa protein as positive controls, FXII-deficient plasma as a negative control, and transferrin as a loading control.

In the 12 adult female participants, there was a significant increase in level of FXIIa protein (a baseline level band density of 29,231 vs 55,201 after 3 months of OCP therapy [*P* < .008]) as well as an increase in level of cHK protein with OCP therapy (*P* = .04) from a baseline band density of 37,820 to 43,797 after 3 months of OCP therapy (Fig 2).

Although the management of HAE has improved significantly in the past 15 years with the approval of C1-INH replacement and therapies targeting BK expression and receptor binding, unmet needs for patients with HAE remain. One unmet need is to better characterize how specific triggers, including estrogen, may exacerbate HAE.

There are data from studies in cardiovascular disease (ie, regulation of blood pressure and circulatory homeostasis) and coagulation, which suggests that estrogen could affect BK expression at several levels, including by decreasing its degradation and regulating the expression of the BK receptor B2 (BKRB2).^10^, ^11^, ^12^ To investigate the potential role of kallikrein production and decidualization, estrogen was administered to ovariectomized animals, resulting in an increase in the concentrations of uterine kallikrein and BK.^15^ Conversely, the administration of progesterone decreased the levels of these mediators. Furthermore, ovariectomy of rats decreased RNA expression of the BKRB2, whereas administration of estrogen increased its expression.^10^ Additionally, administration of an oral estrogen (conjugated equine estrogen combined with a cyclical medroxyprogesterone acetate for 12 days per month) to 19 normotensive postmenopausal women significantly increased serum BK level.^16^

There has been limited work to characterize the mechanisms of how estrogen increases episodes of angioedema in patients with HAE. One potential mechanism to explain increased BK expression with estrogen is that estrogen decreases the activity of AP-P. An analysis of 3 female subjects with estrogen-dependent inherited angioedema with a mutation in factor XII revealed that they also had decreased AP-P levels.^17^ Conversely, women taking oral progesterone contraception had higher age-adjusted plasma AP-P levels.^18^ Drouet et al demonstrated that patients with HAE who were receiving androgen prophylaxis had significantly higher plasma AP-P activity than untreated patients did.^19^ Additionally, several of the published case reports and articles reporting exacerbation of HAE due to OCPs involved subjects using combination estrogen and progestin.^20^^,^^21^

To investigate the connection between estrogen and BK constituents (cHK and FXIIa), we collected plasma before and 3 months after initiation of administration of estrogen-containing OCPs. We performed the study in participants without HAE owing to safety issues of administering estrogen to patients, as estrogen is a known HAE trigger. According to ELISA, the median baseline plasma HK level increased; however, the increase did not reach statistical significance. Of note, the participant taking the highest dose of estrogens had the largest increase in HK level, whereas the participant taking the lowest dose had a decrease in HK level. According to Western blot analysis, expression of both cHK and FXIIa increased. However, it is important to note that our data were generated from a small sample and the analysis was performed on only 2 occasions, which may not have allowed for variation in changes in FXIIa and cHK levels. Nonetheless, our findings are hypothesis generating and require future studies.

A better knowledge of how estrogen may alter BK expression and, hence, exacerbate HAE is of interest for several reasons. First, female patients with HAE may have more severe symptoms than men do.^5^ Second, mechanisms of increased BK expression (including its production and degradation) could be investigated in cases involving other known triggers of HAE, including infection or stress. Third, progesterone may serve as a supplemental prophylactic therapy for female patients with HAE, in particular for those unable to gain access to other medications approved for HAE or those who cannot tolerate other HAE medications. These results suggest that constituents of BK expression may be altered by estrogen-containing OCPs, and therefore, larger studies are needed to characterize this effect.

## Disclosure statement

Supported by Takeda Pharmaceuticals (grant IIR-USA-L00052).

Disclosure of potential conflict of interest: P. J. Busse has received consulting fees and research support from 10.13039/100008322CSL Behring, Kalvista, and Takeda; consulting fees from ADARx, 10.13039/100005276American Academy of CME, Medscape, CVS Caremark, Prime, 10.13039/100014935BioCryst, Pharvaris, Novarits, BioMarin, and Ionis; and consulting fees for expert witness testimony from Hinckley Allen. J. Wisnivesky has received honoraria from 10.13039/100004339Sanofi, Banook, PPD, and Próspero and grants from 10.13039/100004339Sanofi, Arnold Consultant, and 10.13039/100009857Regeneron. The remaining author declares that she has no relevant conflicts of interest.
